# A mask R-CNN model for reidentifying extratropical cyclones based on quasi-supervised thought

**DOI:** 10.1038/s41598-020-71831-z

**Published:** 2020-09-14

**Authors:** Chuhan Lu, Yang Kong, Zhaoyong Guan

**Affiliations:** Key Laboratory of Meteorological Disaster, Ministry of Education/Joint International Research Laboratory of Climate and Environment Change/Collaborative Innovation Center On Forecast and Evaluation of Meteorological Disasters, Nanjing University of Information Science and Technology, Nanjing, 210044 China

**Keywords:** Atmospheric science, Climate change

## Abstract

The applications of machine learning/deep learning (ML/DL) methods in meteorology have developed considerably in recent years. Massive amounts of meteorological data are conducive to improving the training effect and model performance of ML/DL, but the establishment of training datasets is often time consuming, especially in the context of supervised learning. In this paper, to identify the two-dimensional (2D) structures of extratropical cyclones in the Northern Hemisphere, a quasi-supervised reidentification method for extratropical cyclones is proposed. This method first uses a traditional automatic cyclone identification method to construct a trainable labeled dataset and then reidentifies extratropical cyclones in a quasi-supervised fashion by using a (pre-trained) Mask region-based convolutional neural network (Mask R-CNN) model. In comparison, the new method increases the number of identified cyclones by 8.29%, effectively supplementing the traditional method. The newly recognized cyclones are mainly shallow or moderately deep subsynoptic-scale cyclones. However, a considerable portion of the new cyclones along the coastlines of the oceans are accompanied by strong winds. In addition, the Mask R-CNN model also shows good performance in identifying the horizontal structures of tropical cyclones. The quasi-supervised concept proposed in this paper may shed some light on accurate target identification in other research fields.

## Introduction

Extratropical cyclone activity has always been a hot issue because the systems of extratropical cyclones are typically associated with severe storms and intense precipitation; cyclone systems are also closely related to the social economy, especially under the influence of global climate change in recent years. To automatically and reasonably quantify cyclone activity, many researchers have proposed a variety of cyclone identification and tracking algorithms, including the neighbor cyclone center point (NCP) method^1–4^ and the cyclone area algorithm (CAA)^5–9^, based on different perceptions of what best characterizes a cyclone. The CAA can straightforwardly depict the horizontal structure of a cyclone as well as its scale characteristics^8,10^. However, because of the complex structure of an extratropical cyclone, various algorithms still suffer from great uncertainties for moderate and shallow cyclones and for cyclones over mountainous areas^11^.

The concept of deep learning (DL)^12^ was first proposed by Hinton et al.^13^. Since the early twenty-first century, convolutional neural networks (CNNs)^14,15^, one of the classic algorithms for DL models, have achieved remarkable results in the fields of computer vision and image recognition^16^. These successes have provided new ideas for the fields of meteorology and remote sensing^17^. Some experts and scholars have introduced the concept of DL (or CNN models) into their different meteorological research directions^18–25^. For example, Shi et al.^18^ proposed the convolutional LSTM (ConvLSTM) model for precipitation nowcasting based on DL, Hong et al.^19^ used CNNs for typhoon tracking, and Zhang et al.^20^ constructed a CNN model for cloud classification tasks. The automatic identification of the two-dimensional (2D) structure of an extratropical cyclone essentially constitutes the accurate identification of the shape of an object. In recent years, many studies on target recognition have adopted a combination of the region proposal network (RPN) and CNN; as a result, some new models and improved products have been proposed, such as the R-CNN^26^, SPP-Net^27^, Fast R-CNN^28^ and Faster R-CNN^29^. In particular, the Mask R-CNN model, proposed by He et al.^30^, adds a parallel branch to predict the object mask (the object’s spatial profile) based on the Faster R-CNN model (usually used to classify and locate objects) and outperforms existing object pixel-level detection models. Zhang et al.^31^ applied the Mask R-CNN model to the classification and identification of Arctic ice-wedge polygons because of its simple concept and superior capability. The Mask R-CNN model has been shown a good performance in identifying the object-shape in the field of computer vision as well as the geophysics research. This inspires us to apply the model to the identification of the extratropical cyclone, considering the variety and complexity of the structure of extratropical cyclone.

Although DL algorithms have achieved outstanding results (some of which even surpass the human level) on many supervised learning and object identification problems, constructing a large-scale labeled database to train a DL model (such as the Mask R-CNN model) is still a challenging task^32^. Therefore, this paper proposes a method to construct a more reliable labeled dataset for the training of DL models (i.e., the Mask R-CNN model) by using the traditional automatic cyclone identification method instead of a manual labeling scheme. On the other hand, the identification of cyclone extent can effectively reduce the complexity of tracking cyclone-merging or splitting events (e.g., Hewson^33^; Hanley and Caballero^34^). In this paper, first, the CAA proposed by Lu^9^ is used to identify the 2D structure of an extratropical cyclone (north of 20° N), and a 2D extratropical cyclone dataset (quasi-ground-truth) is constructed for training. The Mask R-CNN model is then applied to further identify 2D extratropical cyclones; we refer to this algorithm as an extratropical cyclone quasi-supervised reidentification method. Several previous studies have shown that DL models can provide reliable results when processing complex multi-dimensional meteorological data^18–20^. Our method solves the problem of constructing a large-scale labeled database for DL models by using traditional identifying algorithms, that may efficiently improve the practical efficiency of DL models. Therefore, the performance of the proposed method on extratropical cyclones and its applications in the identification of tropical cyclones are discussed.

## Data and methods

### Data

The data used in this study consist of the ERA-Interim dataset^35^ from 1979 to 2013 (6-h time resolution) with a horizontal resolution of approximately 0.7° × 0.7° (T255 Gaussian grid). Data of the 850 hPa geopotential height field are used to identify extratropical cyclones to the north of 20° N in the Northern Hemisphere.

### Identification process

**Figure 1:**
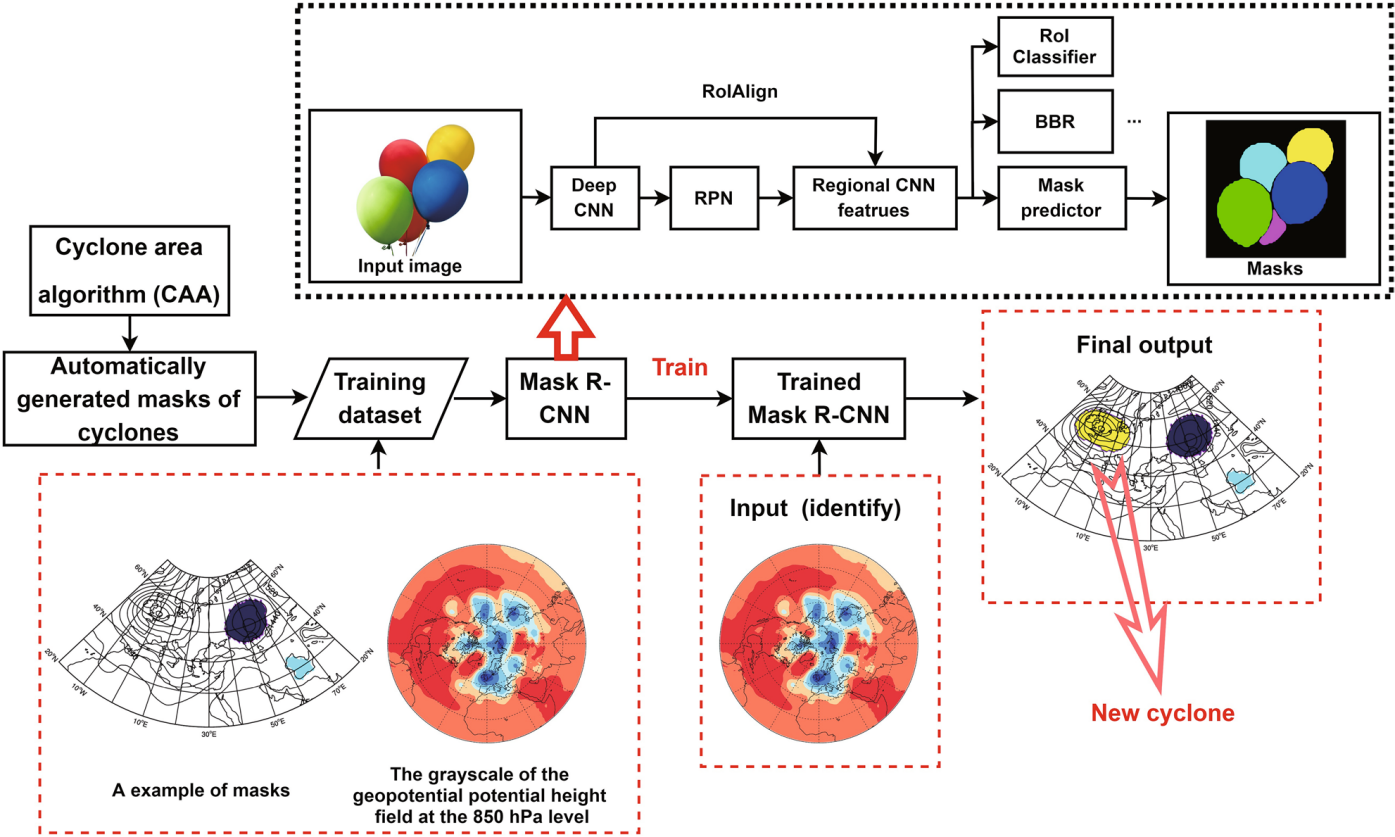
The general workflow of the extratropical cyclone quasi-supervised reidentification method based on the Mask R-CNN model. (The upper black dashed box in the figure shows the simplified basic structure of the Mask R-CNN model, while the red dashed box indicates the input and output).

The general workflow of the extratropical cyclone quasi-supervised reidentification method based on the Mask R-CNN model includes two main steps (Fig. 1). (1) The CAA is used to initially generate the cyclone’s regime (mask); this algorithm simultaneously outputs the boundary of each cyclone, detected by the outermost enclosed contour of the cyclone, while searching for the cyclone center by expanding outward from the center until the outermost enclosed contour is found^9^. (2) Both the cyclone masks output by the CAA and the 850 hPa geopotential height field data are processed as grayscale images to construct a training dataset for the input of the Mask R-CNN model for transfer training. Subsequently, the image of the 850 hPa geopotential height field at each time step is input into the trained Mask R-CNN model, following which the individual mask (consisting of the cyclone’s regime and its peripheral boundary) of each extratropical cyclone at that time can be obtained. Finally, the results of the Mask R-CNN model are supplemented with the CAA results to obtain a combined cyclone identification set. For example, the red dashed box farthest to the right in Fig. 1 shows the contribution of the Mask R-CNN model to the CAA results. The source code of our model is free available on https://github.com/younger-KongY/A-Mask-R-CNN-model-for-identifying-cyclones.

## Results

### Extratropical cyclone identification

Through the method described in identification process, we obtain the identification results of the quasi-supervised reidentifying extratropical cyclone based on the Mask R-CNN model. Over 79% of cyclones in the CAA can be reidentified (with overlapping areas) in the Mask R-CNN model, showing good agreement between the two methods. More importantly, compared with the CAA results, 58,260 individual cyclones are added after being reidentified by the Mask R-CNN model, accounting for 8.29% of the CAA results.

**Figure 2:**
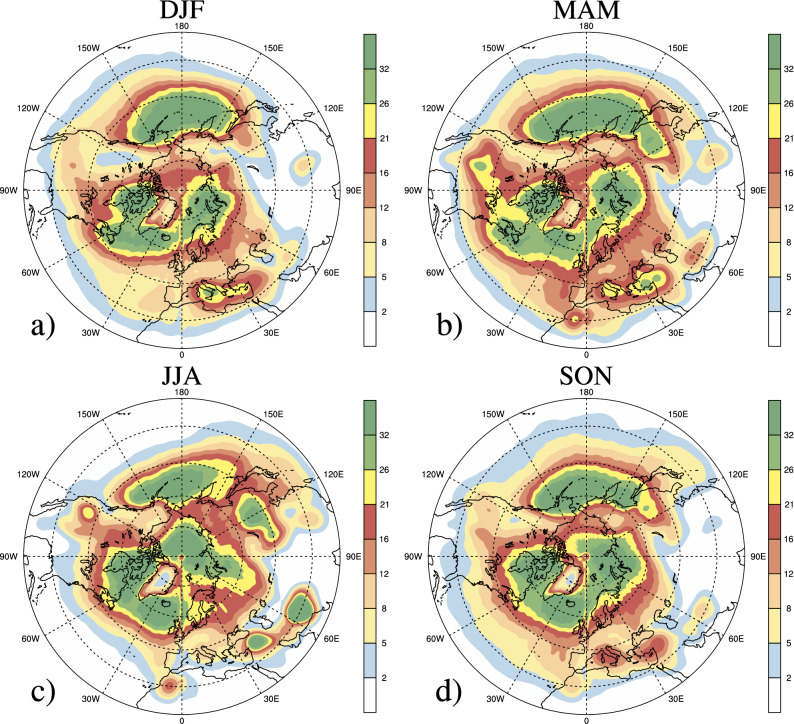
Seasonal mean cyclone 2D-frequencies (%) in the Northern Hemisphere during 1979–2013 for (**a**) DJF, (**b**) MAM, (**c**) JJA, and (**d**) SON.

To further show the robustness of the Mask R-CNN model, we compared the seasonal mean cyclone 2D-frequency (%) with the result in Wernli and Schwierz (cf. their Fig. 4)^7^. The 2D-frequency at every location corresponds to the percentage of time instants that the point is located within a cyclone. As shown in Fig. 2, the seasonal march and spatial distribution of cyclone frequencies derived by Mask R-CNN are identical to Wernli and Schwierz^7^. In particular, high values present over the storm track region in both the North Pacific and the North Atlantic, the Mediterranean as well as Northeast China. The high consistency of results from our model with the ones from Wernli and Schwierz^7^ indicates the robustness of our methodology in this paper. However, our values are comparatively higher than the ones from Wernli and Schwierz^7^. This is because we regard the multi-center cyclone system as one individual cyclone, while a multi-center cyclone system is separated into several cyclones with only one local minimum center in each cyclone in Wernli and Schwierz^7^. Therefore, the values are generally higher in our model.

To illustrate the overall motion characteristics of a newly added cyclone, the ratio of the number of grid points with a positive relative vorticity in the cyclone’s regime to the number of grid points in the entire cyclone mask is defined as the positive vorticity ratio (PVR). Here, the PVR can be expressed as

$$\mathrm{PVR}=\frac{{{\varvec{N}}}_{where(\xi >0)}}{{{\varvec{N}}}_{cyclone size}}.$$

Although high-resolution data of a relative vorticity field can be very noisy^36^, a higher PVR value of a single cyclone generally denotes a stronger counterclockwise rotation of air flow within the cyclone’s area. Therefore, a high PVR represents strong cyclonic motion.

Table 1 shows the distribution of newly added cyclones in different PVR ranges. Most of the cyclones reidentified by the Mask R-CNN model are characterized mainly by counterclockwise rotational motion; among them, 50,416 cyclones (~ 86.54%) are in the range of PVR ≥ 70%. This means that the Mask R-CNN model is good at learning/describing the horizontal structure of an extratropical cyclone and can be used as an effective complement to the CAA. It should be noted that both the CAA and the Mask R-CNN model have a certain proportion of cyclone results with PVR < 50% (0.64% and 1.79%, respectively). As mentioned above, the high resolution of the relative vorticity field could introduce chaotic signals into the cyclone’s regime. Additionally, local terrain or uneven heating could also result in small-scale negative vorticity inside a low pressure system.

**Table 1:** The numbers and ratios of extratropical cyclones identified by the CAA and Mask R-CNN model in different positive vorticity ratio (PVR) ranges.

PVR (%)	0–10%	10–20%	20–30%	30–40%	40–50%	50–60%	60–70%	70–80%	80–90%	90–100%	Total
CAA	842	467	720	930	1,530	6,727	39,791	130,454	197,284	324,056	702,801
	0.12%	0.07%	0.10%	0.13%	0.22%	0.96%	5.66%	18.56%	28.07%	46.11%	100.00%
Mask R-CNN	2	8	40	275	722	1,769	5,028	10,224	13,664	26,528	58,260
	0.00%	0.01%	0.07%	0.47%	1.24%	3.04%	8.63%	17.55%	23.45%	45.53%	100.00%

**Figure 3:**
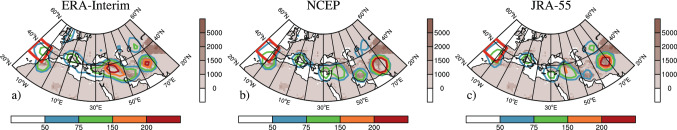
The spatial distribution of newly added cyclones with PVR ≥ 70% in the western and central regions of Eurasia (WCE). The red box marks the coastal region of Western Europe (20°–8° W, 32°–42° N). (**a**) ERA-Interim; (**b**) NCEP; (**c**) JRA-55.

The newly added cyclones are located mainly in the western and central regions of Eurasia (WCE, 20° W–80° E, 20°–60° N), accounting for 35.6% of all newly identified extratropical cyclones. As shown in Fig. 3a, areas with large proportions of newly added cyclones are found in the mountains of WCE (the Armenian Plateau, Zagros Mountains, and Hindu Kush Mountains) and the Atlas Mountains in northwestern Africa, while the areas with the second-highest proportions are distributed mainly along the oceanic coastlines (for example, along the coastlines of Western Europe and the Mediterranean Sea). On the other hand, the supplements along the two major storm tracks in the North Pacific and the North Atlantic are inconspicuous (figure not shown). This is probably because these storm track regions are located mainly on the ocean surface, resulting in a relatively symmetrical cyclone shape. Both the Mask R-CNN model and CAA can identify these cyclones well, and the supplementary effect of reidentification is not notable. We also applied the Mask R-CNN to the cyclone identification with NCEP I and JRA55. Consistently, the cyclone frequency in NCEP I and JRA 55 agree well with ERA-interim. WCE is still the most newly cyclone-added regions (43.7%, 39.2% for NCEP I and JRA55 respectively). Furthermore, the spatial distributions of newly added cyclones with PVR ≥ 70% in NCEP I and JRA 55 are also identical to ERA-interim (Fig. 3b,c). Hence, the following analysis of these newly identified cyclones will focus on WCE based on ERA-interim reanalysis dataset.

**Figure 4:**
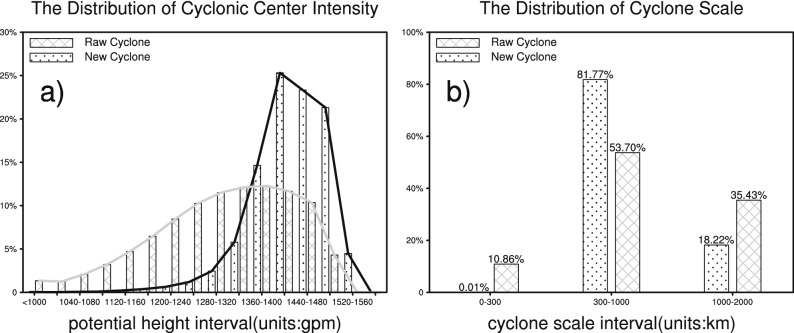
The characteristics of cyclones (PVR ≥ 70%) identified by the Mask R-CNN model over the western and central regions of Eurasia (WCE) and the CAA: (**a**) cyclonic center intensity and (**b**) cyclone scale (the equivalent circle diameter of cyclone).

Compared to the identified cyclones in the CAA results, the newly added cyclones in WCE are relatively weak. As shown in Fig. 4a, a large proportion (84.60%) of the minimum geopotential heights of cyclones (denoting their intensity) in the Mask R-CNN model results are in the range of 1,360–1,520 gpm. Comparatively, a greater number of intense cyclones detected by the CAA are in the range of 1,160–1,480 gpm. The diameter of a regular circle is treated as the equivalent horizontal extent of the cyclone. As shown in Fig. 4b, both the Mask R-CNN model and the CAA focus mainly on the detection of cyclones with a subsynoptic scale (300–1,000 km) and a synoptic scale (1,000–2,000 km). However, the proportion of newly added cyclones at the subsynoptic scale identified by the Mask R-CNN model is notably higher than that identified by the CAA. Therefore, the new cyclones identified by the Mask R-CNN model are mostly located at the shallow or moderate subsynoptic scale. Note that, most of the newly added cyclones occurred in the Iranian Plateau and west of Tibet Plateau, and the cyclones identified over high terrain are generally shallow, which satisfies common sense.

**Figure 5:**
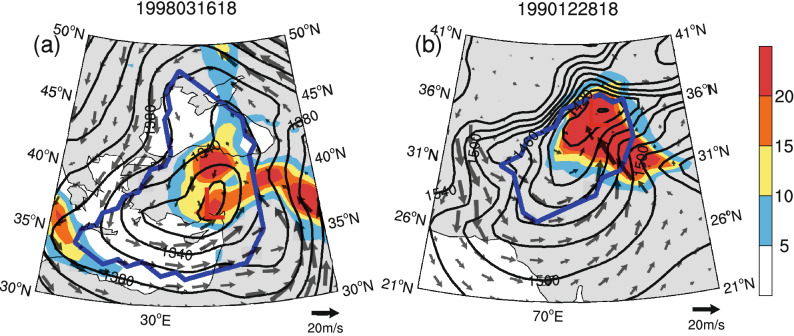
The strongest newly added cyclones identified over high terrain (> 1,500 m). The thick blue lines indicate the cyclone’s boundaries, the gray arrows indicate wind vectors (units: m/s), and the shading indicates the 24-h total precipitation (units: mm/day).

Figure 3 shows that most newly added cyclones occur predominantly over mountainous areas, which may be due to the filtering out of terrain at high elevations (> 1,500 m) and the Tibetan Plateau region (20°–45° N, 65°–110° E) in the CAA^9^. In fact, different traditional automatic cyclone identification algorithms suffer from great uncertainties in identifying cyclones over mountainous areas because they deal with mountains using different strategies^11^. To alleviate the influences of local artificial lows and reduce the complexity of the algorithm, some algorithms directly filter out mountainous terrain with different thresholds. However, we found that 14.28% of newly identified cyclones with PVR ≥ 70% in WCE are located in high-elevation mountainous areas (> 1,500 m). It is worth noting that simply filtering out high-elevation terrain may ignore some high-impact extratropical cyclones over such mountainous areas. For example, Fig. 5a,b show two of the strongest newly added cyclones identified over high terrain (> 1,500 m). These cyclones are accompanied by clearly cyclonic winds and distinct local precipitation. Therefore, the Mask R-CNN model could be an effective way to objectively detect extratropical cyclones over mountains.

**Figure 6:**
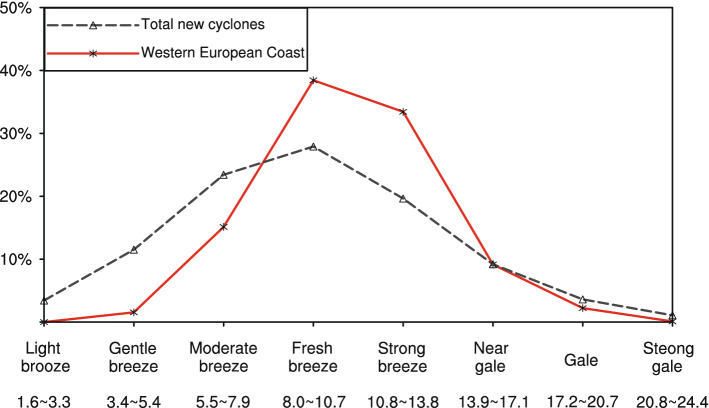
The maximum 6-h-mean 1,000 hPa wind speeds (units: m/s) of newly identified cyclones in the coastal region of Western Europe (20°–8° W, 32°–42° N) and all new cyclones with PVR ≥ 70%.

In addition to mountainous cyclones, many new cyclones are identified near the coastlines of the oceans. These coastal cyclones could cause severe winds and other disasters (e.g., Pinto and Silva^37^). For example, the new cyclones in the coastal region of Western Europe (20°–8° W, 32°–42° N, red box in Fig. 3) are accompanied by relatively high wind speeds (Fig. 6). In particular, although these cyclones are mostly subsynoptic-scale cyclones (approximately 89.02%), the maximum 6-h-mean 1,000 hPa wind speeds of 44.9% of all coastal cyclones are over 10.8 m/s (above the level of a strong breeze). Since the meso- or subsynoptic-scale cyclones are usually with short-lived life cycles, they are generally filtered to omit some of the local heat lows. However, because of relatively high wind speeds related to the coastal cyclones, the spatiotemporal variation of newly added cyclones in the coastal region of Western Europe deserved further investigation.

**Figure 7:**
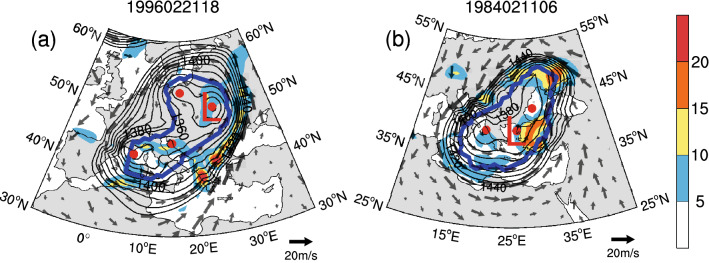
The strongest newly added multicenter cyclones (MCCs) with the largest horizontal scale. The thick blue lines indicate the cyclone’s boundaries, the gray arrows indicate wind vectors (units: m/s), and the shading indicates the 24-h total precipitation (units: mm/day).

The nearest-neighbor method is widely applied to track cyclones. However, it is difficult for the nearest-neighbor method to detect cyclones when more than two points in a particular time frame become merged into a single point in the following time frame (or vice versa). Therefore, some methods for the identification of multicenter cyclones (MCCs) have been proposed to detect the merging and splitting of cyclones (e.g., Inastu^8^; Hanley and Caballero^34^; Lu^9^). Among the newly detected cyclones with PVR ≥ 70% in WCE, 42.21% of them are MCCs. Figure 7a,b display the two strongest new cyclones with the largest horizontal scale; both cyclones clearly show multicenter structures. Furthermore, both MCCs have a uniform overall cyclonic circulation accompanied by obvious local precipitation.

### Tropical cyclone (TC) identification

Since the Mask R-CNN model also has great portability, we further apply this model to identify the 2D structures of tropical cyclones (TCs); the model is extended in comparison with Hong et al.^19^, who employed CNNs to track the eyes of typhoons. According to the typhoon track dataset from the China Meteorological Administration (CMA) in the western Pacific (20° S–65° N, 100°–180° E), we manually locate the center of each typhoon and its corresponding regime (the area inside its outermost enclosed contour) in the 6-hourly sea level pressure (SLP) field. Accordingly, a trainable 2D TC mask (labeled) dataset is constructed from 2011 to 2018. The Mask R-CNN model is trained by using the dataset from 2011 to 2015 to equip itself with the ability to identify the 2D structures of TCs. Finally, the SLP dataset from 2016 to 2018 is utilized to validate the ability of the Mask R-CNN model to identify TCs.

**Figure 8:**
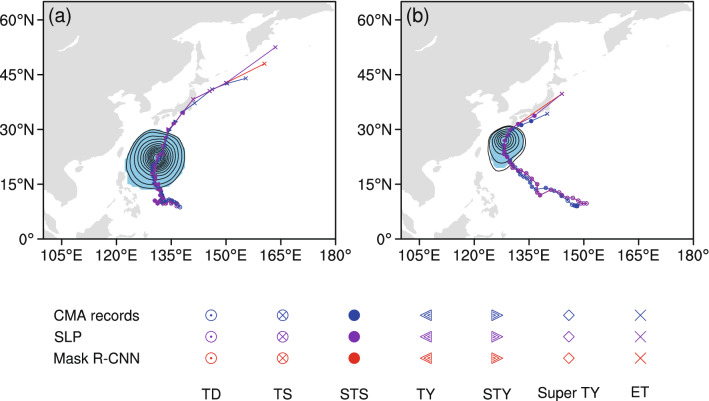
Two examples of typhoon events identified by the Mask R-CNN model. (**a**) Typhoon No. 24 in 2017; (**b**) typhoon No. 25 in 2017. The blue shading indicates the identified 2D structure of the TC at its strongest time frame.

To evaluate the performance of the Mask R-CNN model in identifying TCs from 2016 to 2018, the matching rate is defined as the ratio of the number of TCs identified by the Mask R-CNN model to the total number of TCs within the SLP field. As documented in Table 2, the matching rate of tropical depressions (TDs) is 78.79%. As the intensity of the TC becomes stronger, the matching rate exceeds 90%. Taking the lowest pressure in the identified TC area as the center of the TC, Fig. 8 shows the tracks and intensities of typhoon events No. 24 and No. 25 in 2017 based on the Mask R-CNN model. In these two cases, the tracks of these two TCs and their 2D structures are highly consistent with the manually identified typhoon tracks based on the SLP dataset and are also in good consistency with the CMA typhoon track records. Based on the above results, the transfer learning capability of the Mask R-CNN model could be effectively applied to objectively identifying the 2D structures of TCs. Note that the TC extend is an arguably realistic and reliable quantification of the system strength. This characteristic can be used to study the local physical relationship between cyclones and extreme precipitation^38,39^.

**Table 2:** The matching rate for identifying TCs from the SLP field in the western Pacific during 2016 to 2018.

Intensity category	Tropical depression	Tropical storm	Severe tropical storm	Typhoon	Severe typhoon	Super typhoon	Total
SLP	495	567	247	235	185	144	1,873
Mask R-CNN	390	514	236	235	184	143	1,702
Matching rate	78.79%	90.65%	95.55%	100.00%	99.46%	99.31%	90.87%

## Conclusions

Massive amounts of meteorological data are conducive to improving the training effect and model performance of ML/DL, but constructing a large-scale labeled database to train a ML/DL model is still a challenging task, especially in the context of supervised learning. The construction of training datasets is usually approached in a manual way, which is time consuming for a large number of samples. In this paper, a quasi-supervised reidentification method for extratropical cyclones is proposed. This method first uses the CAA to construct a trainable labeled dataset and then reidentifies the 2D structures of extratropical cyclones in a quasi-supervised fashion by using a (pre-trained) Mask R-CNN model.

We found that our quasi-supervised reidentification method for extratropical cyclones based on the Mask R-CNN model adds 58,260 new cyclones from 1979 to 2013. As measured by their PVR values, most of these new cyclones display obvious counterclockwise rotational motion characteristics, with 86.54% of these cyclones having PVR ≥ 70%. These new cyclones are located mainly (~ 35.6%) in the mountainous areas of WCE, including the Atlas Mountains in northwestern Africa and along the coastlines of Western Europe and the Mediterranean Sea. Approximately 81.8% of all newly added cyclones are subsynoptic-scale cyclones, most of which are shallow or moderately deep, and 14.28% of the new cyclones are situated above high-elevation (> 1,500 m) mountainous areas. In addition, we found that the new cyclones affecting the coastal areas of Western Europe mostly occur at the subsynoptic scale but with relatively strong wind speeds and potentially high impacts for these areas.

The quasi-supervised method based on the Mask R-CNN model also has a good transfer learning ability for identifying TCs. In particular, the Mask R-CNN model can effectively capture the track and 2D structure of a TC (in comparison with manual identification approaches) with a matching rate above 90%. Therefore, the quasi-supervised concept proposed in this paper may shed light on target recognition tasks in other research fields by using classic automatic algorithms for the construction of training datasets to improve the practical efficiency of ML/DL and the reliability of object recognition.
